# Comparative Assessment of Grassland Dynamic and Its Response to Drought Based on Multi-Index in the Mongolian Plateau

**DOI:** 10.3390/plants11030310

**Published:** 2022-01-25

**Authors:** Yanzhen Zhang, Zhaoqi Wang, Qian Wang, Yue Yang, Yaojun Bo, Weizhou Xu, Jianlong Li

**Affiliations:** 1 College of Life Sciences, Yulin University, Yulin 719000, China; zhangyanzhen@yulinu.edu.cn (Y.Z.); byj212@123.com (Y.B.); 2 Department of Ecology, School of Life Science, Nanjing University, Nanjing 210023, China; 3 State Key Laboratory of Plateau Ecology and Agriculture, Qinghai University, Xining 810016, China; wangzhaoqi_818@163.com; 4 School of Environment and Planning, Liaocheng University, Liaocheng 252059, China; qianwang@lcu.edu.cn; 5 Nanjing Institutes of Environmental Sciences, Ministry of Environmental Protection of the People’s Republic of China, Nanjing 210042, China; yangyue@nies.org

**Keywords:** the Mongolia Plateau, grassland degradation, vegetation coverage, surface bareness degree, self-calibrating Palmer Drought Severity Index (scPDSI)

## Abstract

This study applied grassland related multi-index and assessed the effects of climate change by investigating grassland responses to drought. This process was performed to study grassland vegetation dynamic accurately and evaluate the effect of drought in the Mongolian Plateau (MP). The spatial–temporal characteristics of grassland dynamic in terms of coverage (F_v_), surface bareness (F_b_), and net primary production (NPP) from 2000 to 2013 were explored. We implemented the maximum Pearson correlation to analyze the grassland vegetation in response to drought by using self-calibrating Palmer Drought Severity Index (scPDSI). Results show that F_v_ and NPP present an increasing trend (0.18 vs. 0.43). F_b_ showed a decreasing trend with a value of −0.16. The grassland F_v_ and NPP positively correlated with scPDSI, with a value of 0.12 and 0.85, respectively, and F_b_ was −0.08. The positive correlation between F_v_ and NPP accounted for 84.08%, and the positive correlation between F_v_ and scPDSI accounted for 93.88%. On the contrary, the area with a negative correlation between F_b_ and scPDSI was 57.43%. The grassland in the MP showed a recovery tendency. The increase in grassland caused by positive reaction was mainly distributed in the middle of Mongolia (MG), whereas that caused by counter response was mainly distributed in the east and west MG and northeast Inner Mongolia autonomous region of China (IM). The relevant results may provide useful information for policymakers about mitigation strategies against the inverse effects of drought on grassland and help to ease the losses caused by drought.

## 1. Introduction

As the earth’s largest terrestrial ecosystem, grassland plays an important role in ecosystem cycles [1,2,3]. Evaluating the dynamic change in grassland ecosystem quantitatively is urgent because grassland provides many economic products and ecological services [4,5]. Previous research investigated the impact of climate change on the grassland vegetation dynamic by using different indicators. The indicators to evaluate grassland vegetation dynamic by remote sensing technology mainly include the normalized difference vegetation index (NDVI), enhanced vegetation index (EVI), vegetation coverage (F_v_), and net primary productivity (NPP) [6,7,8]. Some recent studies have proposed ground bareness (F_b_) as another important parameter of global land cover change [9,10]. As an opposite concept of F_v_, F_b_ contains the attribute of surface reflectivity and temperature information of grassland vegetation rather than a complementary set of coverage. In recent years, research has focused on drought events by using the combination of identified NPP and NDVI [11]. Compared with single index analysis, the vegetation dynamic inversion based on multi-index can help to improve the reliability of results due to the diversities of analysis.

Drought is a natural phenomenon where the availability of water is significantly lower than normal for a long period and the supply cannot meet the existing demand [12]. With global warming, drought is quickly becoming a devastating environment incident [13]. The International Disaster Database estimated that droughts attributed approximately 5% of the natural disasters over the globe, and the losses caused by drought disasters accounted for more than 30% of those of the natural hazards [14]. Drought has been a crucial scientific issue in the domain of climate research due to its negative effects on water resources, livestock husbandry development, and local economy [15,16,17]. The influence of drought on terrestrial ecosystems is becoming increasingly acute [18]. The grassland dynamics and its response to driving factors are always investigated by researchers because grassland is more susceptible to droughts than other ecosystems [19]. Previous studies explored the impact of drought on grassland vegetation dynamic at multiple regions. Some researchers evaluated the NPP distribution and response to drought in Europe [20]. Their results suggest that rainfall deficit and extreme summer heat reduce the vegetation productivity in Eastern and Western Europe, respectively. Another study strengthened the conclusion of drought-induced reduction in NPP over the past decade in central Asia [21]. Therefore, a better understanding in grassland vegetation dynamic and its feedback on climate change will improve the local economic development, especially for the typical farming and pastoral areas.

The Mongolian Plateau (MP) is a typical arid and semiarid area, with natural grassland as the dominant vegetation type. It often suffered from different conditions of drought due to the decreasing water resource supply and climate change [22,23]. Droughts over the last century induced a heap of negative effects, such as water resource shortages, threat of food shortages, and vegetation degradation [24,25,26,27]. Therefore, quantitative assessment of grassland vegetation dynamic and the effect of droughts is urgent. In accordance with the recent analysis, the summer drought has contributed to the increasing extreme droughts since the 1990s [28]. Some researchers have proven that the self-calibrating Palmer Drought Severity Index (scPDSI) is suitable than other drought indexes when considering the impact of precipitation and temperature on the soil moisture in Inner Asia [29]. Another research from Wang revealed that the global grassland scPDSI value has a slightly increasing trend with a rate of 0.0119 per year [30]. However, there is still a lack of research on grassland vegetation dynamic and its response to droughts of the MP.

There are many related previous studies focused on single or two vegetation indexes to evaluate the grassland dynamic and its response to climate factors [31,32,33]. To enrich vegetation related research indicators, we selected F_v_, F_b_, and NPP to reflect the grassland vegetation dynamic for improving the reliability of conclusions. We evaluated the grassland response to droughts during the study period. A combined analysis of the three indexes in different drought severity areas was quantitatively assessed to enhance the credibility of the results. The results may provide a scientific basis for guiding ecological environment improvement and drought prevention for typical farming and pastoral areas in the world. 

## 2. Results

### 2.1. Spatial and Temporal Distribution of F_v_, F_b_, and NPP

**Figure 1:**
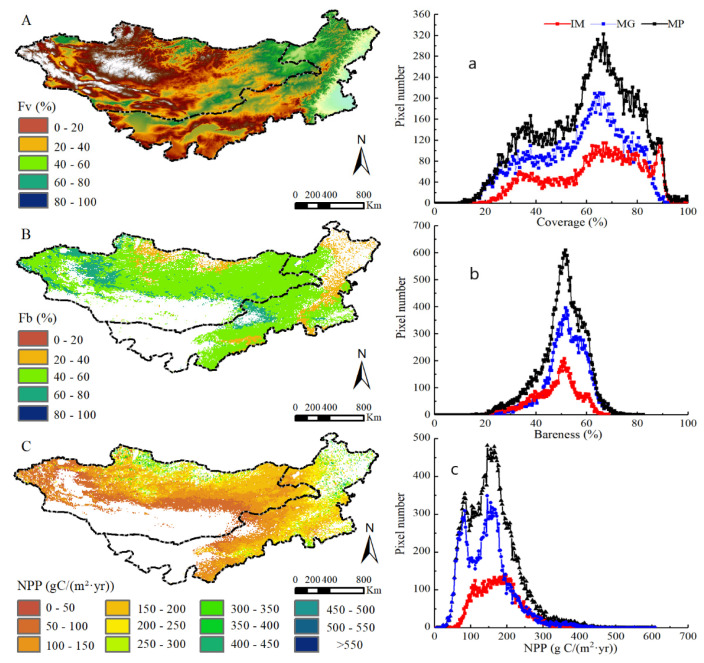
Spatial distribution of grassland F_v_, F_b_ and NPP and statistics of the corresponding pixel number during 2000–2013. ((**A**–**C**) are the spatial distribution of F_v_, F_b_ and NPP, respectively; (**a**–**c**) is the statistics of the corresponding pixel number).

The spatial distribution of long-term mean grass F_v_, F_b_, and NPP in the Mongolia Plateau is shown in Figure 1. The grass F_v_ value is relatively higher in northern and northeastern MP, while lower in southwestern and western MP (Figure 1A). On the contrary, F_b_ greater than 60% distributed over the southwestern and western MP, while F_b_ less than 40% mainly distributed over the northeastern and northern MP (Figure 1B). The mean actual NPP showed obvious spatial heterogeneity, too (Figure 1C). Areas with mean actual NPP larger than 200 g C/(m^2^·yr) were scattered in the northern and northeastern MP with good vegetation growth conditions. Areas with mean actual NPP lower than 100 g C/(m^2^·yr) were mainly scattered in the regions with relatively scarce water resources and vegetation in the transition area of grassland and desert such as southwestern and western MP. We counted the different pixel values of grassland F_v_ (Figure 1a), F_b_ (Figure 1b), and NPP (Figure 1c) in the MP. The average F_v_, F_b_, and NPP values were 18.42%, 15.53%, and 61.41 g C/(m^2^·yr), respectively, whereas the corresponding distribution rates of their peak value were 60–80%, 40–60%, and 150–200 g C/(m^2^·yr). The F_v_, F_b_, and NPP of IM were 9.17%, 6.84%, and 24.82 g C/(m^2^·yr), respectively. MG had higher values of the three indexes than IM. The corresponding distribution rates of IM and MG peak values were similar to the MP. 

**Figure 2:**
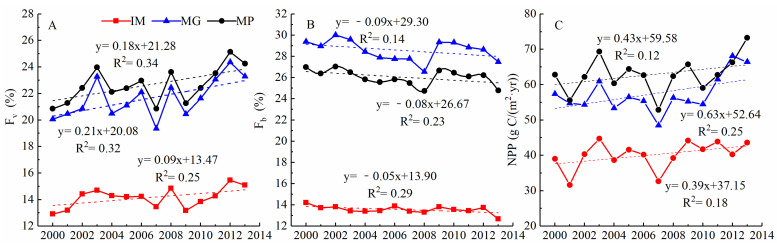
The statistics of the temporal distribution of grassland F_v_, F_b_, and NPP. ((**A**–**C**) are the statistics of F_v_, F_b_, and NPP during 2000–2013, respectively).

In this study, grassland F_v_ in the MP exhibited an increasing trend from 2000 to 2013, with a 14-year cumulative increment of 0.18 (Figure 2). MG had a higher F_v_ value than IM (0.21 vs. 0.09). On the contrary, grassland F_b_ showed an overall decreasing trend in the MP, with the decreased rate of −0.08. The decrease rates of MG and IM were −0.09 and −0.05, respectively. NPP had the largest change rate compared with the two other indexes, with a 14-year cumulative increment of 0.43. MG had a higher increase value than IM (0.63 vs. 0.39).

### 2.2. Dynamic Analysis of Grassland

**Figure 3:**
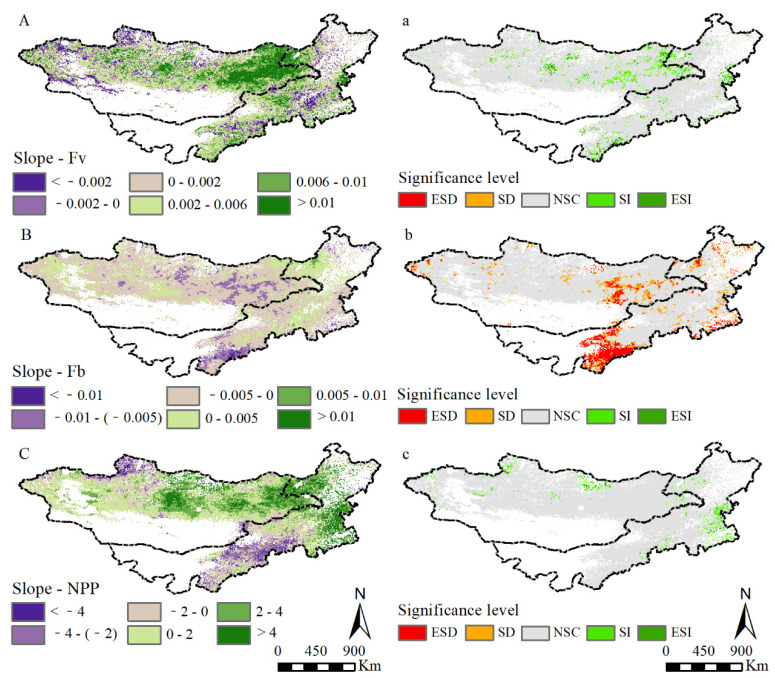
The changing trend and significance levels of grassland F_v_, F_b_ and NPP. ((**A**–**C**) are the changing trend of grassland F_v_, F_b_ and NPP, respectively. (**a**–**c**) are the corresponding significance levels. ESD (extremely significant decrease, slope < 0, *p* < 0.01); SD (significant decrease, slope < 0, 0.01 < *p* < 0.05); NSC (no significant change, slope = 0, *p* > 0.05); SI (Significant Increase, Slope > 0, 0.01 < *p* < 0.05); ESI (extremely significant increase, slope > 0, *p* < 0.01)).

The changing trend and significance levels of grassland F_v_, F_b_, and NPP in the MP from 2000 to 2013 are shown in Figure 3. The growth rate of F_v_ occupied 60.51% of the MP grassland, mainly found in the east and central MG, east Xing’an, south Ordos, and central IM (Figure 3A). On the contrary, the regions of F_b_ exhibiting decreasing trends were extremely larger than that with increasing trends (92.64% vs. 7.36%), with the decreased rate of −0.0005/14a. The decreased regions were mainly found in the entire MP, typically occurring in the southwest and middle MP (Figure 3B). The NPP increasing areas occupied 79.54% of the MP grassland, mainly found in Kent Mountains and Hanggai Mountains in MG and east Xing’an League in IM (Figure 3C). F_v_ with clear increases was distributed in the east Dornod, Hangai Mountains, and Kent Mountains in MG, and east Xing’an and south Ordos in IM (Figure 3a). F_b_ exhibited a significant decrease (SD) and an extremely significant decrease (ESD), accounting for 14.77% and 11.61% of the MP grassland, respectively (Figure 3b). The regions of NPP with a significant increase (SI) accounted for 4.27% of the MP grassland. The regions with significant increase were mainly distributed in the east Selenge in MG and east Xing’an in IM (Figure 3c).

### 2.3. Correlation Analysis of Grassland Indexes to scPDSI

**Figure 4:**
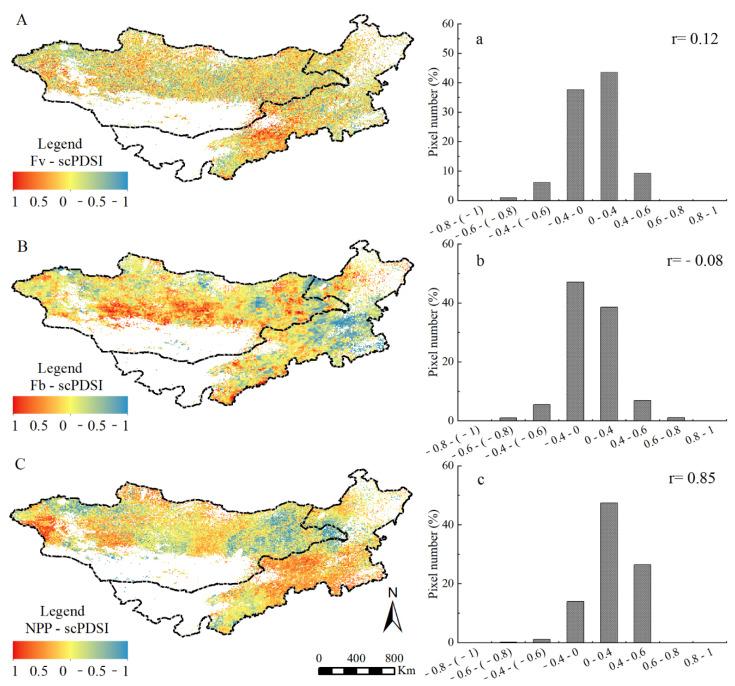
The spatial distribution and pixel frequency of correlation coefficient between scPDSI and three vegetation indicators ((**A**–**C**) are the correlation coefficient spatial distribution and (**a**–**c**) corresponding its pixel frequency).

The correlation coefficient of grassland indexes and scPDSI was analyzed because grassland dynamic is driven by global climate change (Figure 4). F_v_ and NPP were positively correlated with scPDSI, with a value of 0.12 and 0.85, respectively, whereas F_b_ accounted for −0.08. The areas with a positive correlation between F_v_, NPP, and scPDSI were approximately 84.08 and 93.88%. On the contrary, a negative correlation between F_b_ and scPDSI accounted for 57.43%. The grassland regions (2.02%) showed a significant positive correlation (*p* < 0.05) between F_v_ and PSDI, mainly distributed in Baotou, Hohhot, and south Ulaan Chab in IM. The grassland areas (8.28%) showed significant negative correlation (*p* < 0.05), mainly distributed in the Kent mountain area of MG and Tong Liao, Chi Feng, and Xilin Gol of IM. In the regions with a positive correlation between NPP and PSDI, 19.57% of them showed a significant positive correlation (*p* < 0.05), mainly distributed over the west, north, and central MG, and central IM.

### 2.4. Changes and Trends in Grassland Response to Drought

**Figure 5:**
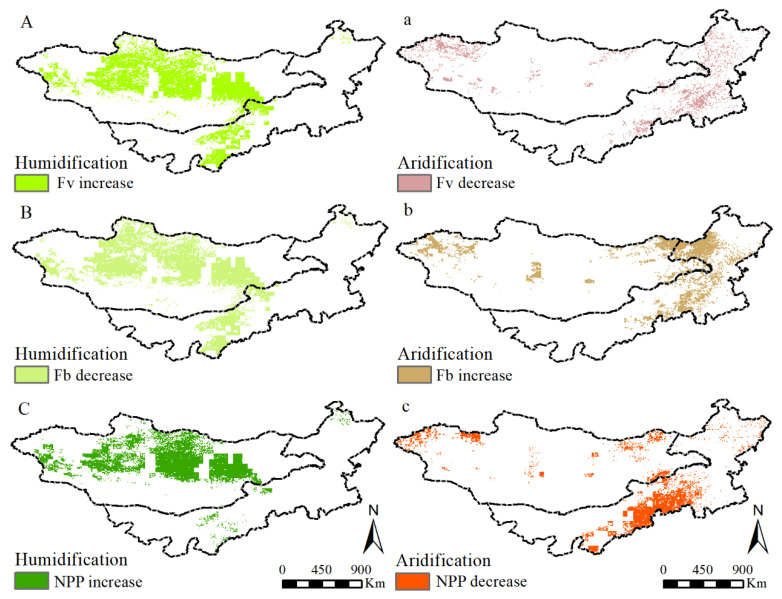
The control response of F_v_, F_b_, and NPP to PDSI changes in the Mongolian plateau from 2000 to 2013. ((**A**–**C**) are the correlation coefficient spatial distribution and (**a**–**c**) corresponding its pixel frequency).

**Figure 6:**
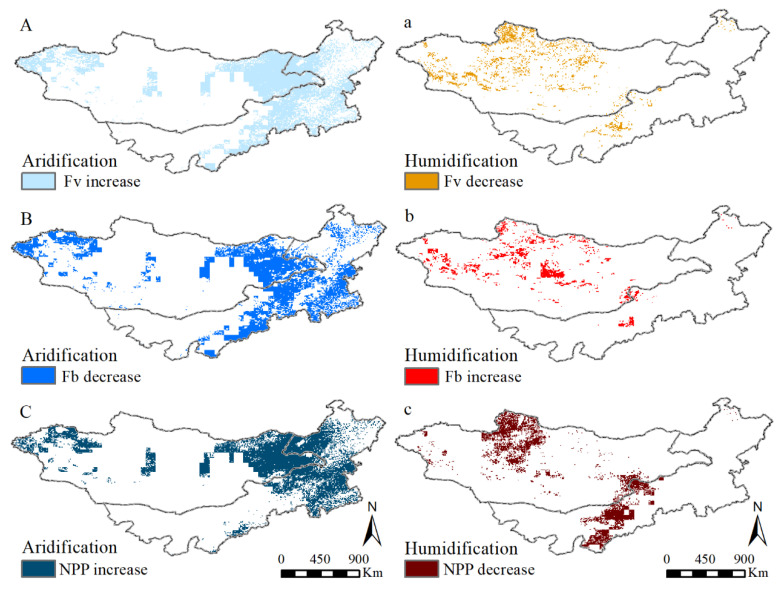
The counter response of F_v_, F_b_, and NPP to PDSI changes in the Mongolian plateau from 2000 to 2013. ((**A**–**C**) are the correlation coefficient spatial distribution and (**a**–**c**) corresponding its pixel frequency).

The area of grassland F_v_, F_b_, and NPP responding to scPDSI in the control response for grassland increase accounts for 36.55, 37.99, and 28.23% of the total area, respectively (Figure 5A–C). The control response to grassland increase from F_v_, F_b_, and NPP to scPDSI appear in similar areas, mainly concentrating on central, north, and west MG, and west IM. On the contrary, the control response to grassland decrease accounts for 10.09, 17.10, and 14.98% (Figure 5a–c). It is mainly concentrated on the south Sayan Mountains, south Hangai Mountains, and Dornod in MG, and northeast and south IM. The area of grassland F_v_, F_b_, and NPP responding to scPDSI in the counter response for grassland increase accounts for 44.73, 37.76, and 40.34% of the total area, respectively (Figure 6A–C). The counter response to grassland increase from F_v_, F_b_, and NPP to scPDSI appear in similar areas, mainly concentrating on the northeast and west MG and northeast and south IM. On the contrary, the counter response to grassland decreases accounts for 8.63, 7.15, and 16.45% (Figure 6a–c). It is mainly concentrated on central MG and west IM. 

## 3. Discussion

### 3.1. Methodology

The current study used the slope-combined analysis based on multi-index to simulate grassland vegetation dynamic and monitor grassland response to droughts. The hypothesis is that grassland F_v_ and NPP dynamic are a positive feedback, whereas F_b_ is on the contrary. Previous studies applied single index, such as NDVI, F_v_, and NPP, to simulate the grassland dynamic. However, many uncertainties remain due to the inversion model or the uncertainty of dataset itself [34]. The advantage of the current method is the reference of F_b_ index. Our findings show that 12.93% of the grassland in the MP experiences an increasing trend compared with 0.73% of the grassland that experienced a decreasing trend during the study period. Several studies about grassland NPP showed that grassland has an increasing trend in the similar area during the study period [35,36]. Similarly, studies on vegetation indexes, such as NDVI, F_v_, and EVI, show an increasing trend of grassland vegetation [37,38,39]. Thus, the present studies confirmed that the grassland shows a recovery trend in the MP, which agrees with our findings.

### 3.2. Climate Factors on Grassland Vegetation Dynamic

**Figure 7:**
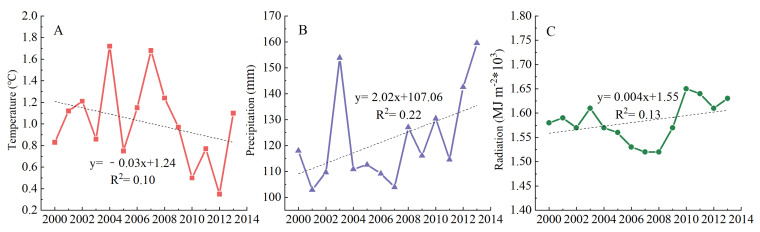
Temporal variations of meteorological variables during 2000–2013 ((**A**–**C**) are the annual mean temperature, annual cumulative precipitation, and annual cumulative solar radiation, respectively).

In this study, we assessed the grassland dynamic on the basis of F_v_, F_b_, and NPP and their impact on droughts during 2001 to 2013. The results provided a new understanding of drought-driven grassland change in the MP. Climate variations, such as temperature and precipitation, influenced terrestrial vegetation directly. These climate factors regulated soil respiration, photosynthesis, growth status, and distribution [40]. Here, we calculated the temporal trends of temperature, precipitation, and radiation during the study period (Figure 7). The temperature in this study showed a downward trend (−0.03 °C), whereas precipitation and radiation showed an increasing trend (2.02 mm and 3.39 MJ/m^2^) due to the short study period. Evidence shows that global warming is definitely occurring, and the climate in our study area tended to be wet and warm [41]. Typically, the combination of warmer temperature and higher precipitation concentration during the early growing season possibly increased NPP, partly by lengthening the growing season [11]. The related research shows that the carbon sequestration capacity of grassland ecosystem is enhanced by increased precipitation, which supports our findings [42,43]. Another study revealed that global warming helps to increase the productivity and carbon storage of grasslands in China [34]. 

### 3.3. The Role of Ecological Policies in Grassland Restoration

**Figure 8:**
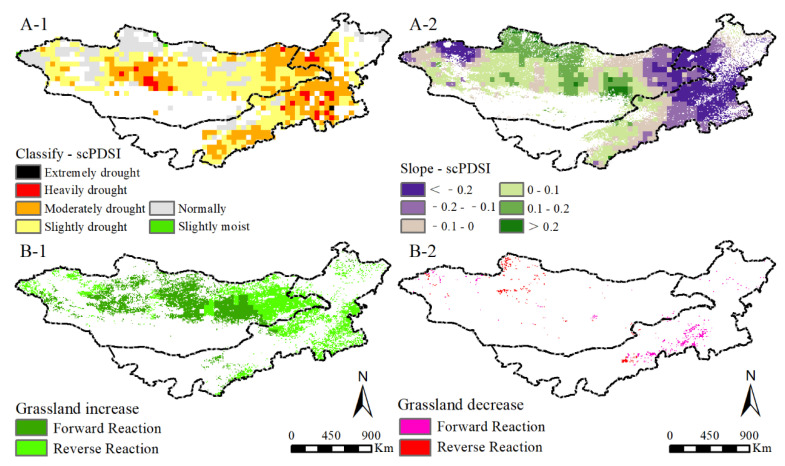
The spatial distribution (**A-1**) and changing trend (**A-2**) of scPDSI and the reaction of F_v_, F_b_, and NPP to scPDSI changes (**B-1**,**B-2**) in the MP from 2000 to 2013.

As a limited resource, water is necessary for plant growth and development, especially in arid and semi-arid ecosystems [44]. Evidence showed that grasslands experience different degrees of drought in the MP (Figure 8A-1). Although drought associates with decreased precipitation, increased precipitation does not necessarily weaken the drought [45,46]. A slight reduction of drought is observed in the MP (−0.02), mainly concentrating in the western and eastern MG (Figure 8A-2). This finding is consistent with other studies that used SPI and SPEI to show drought [47,48,49]. We fitted the response from grassland F_v_, F_b_, and NPP to scPDSI, and the results showed a recovery trend (Figure 8B-1,B-2). Grassland increased regions are obviously larger than the decrease regions (12.93% vs. 0.73%), strongly confirming the recovery of grassland in the MP. The grassland increase regions with control response to drought mainly distributed in the central MP. Few human activities were found in these areas, and the vegetation growth was mainly affected by natural climatic factors [50]. The grassland increase regions with counter response to drought were in the eastern and western MG and northeast IM. This finding shows that other factors, such as human activities affecting local grassland restoration, are greater than the climate factors [51]. The distribution of grassland decrease regions with control response and counter response is minimal (0.44% vs. 0.29%). The grassland decrease regions with control response mainly distributed in south central IM and north MG. This finding reveals the grassland degradation in these areas are under complex influence factors, such as increase pressures from people and livestock populations [52].

## 4. Materials and Methods

### 4.1. Study Area

**Figure 9:**
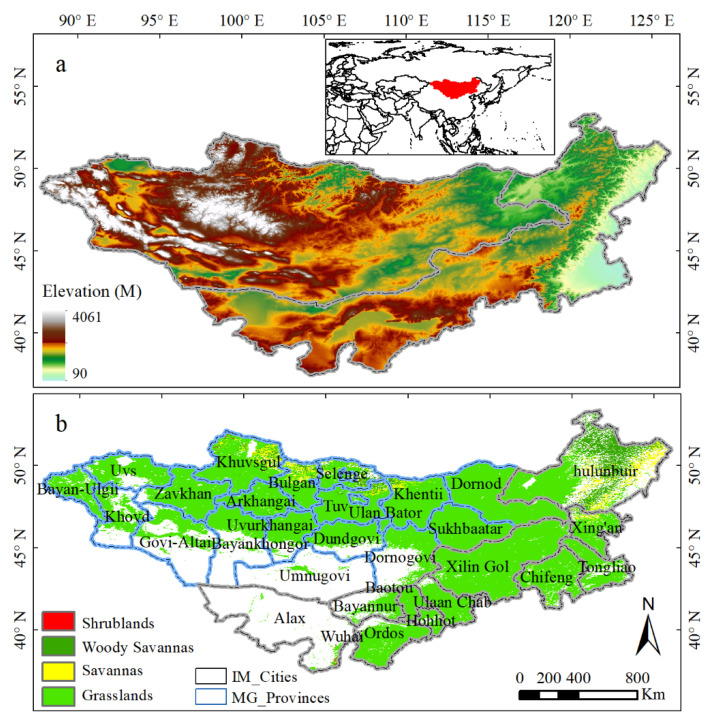
Elevation of the MP (**a**) and grassland types derived from MODIS land cover product (MCD12Q1, IGBP) by the year of 2013 (**b**).

The MP is located in Siberia in the north to the northern Yinshan in the south, from the Outer Xing’an Mountains in the east, to the Altai Mountains in the west (37°22′–53°20′ N and 87°43′–126°04′ E, Figure 9). The Altai Mountains in the southwest, the Kent Mountains in the north, and the outreach area of the Xing’an Mountains in the east are found in the study area, with the mean elevation of 1580 m (Figure 9a). The MP mainly includes Mongolia and Inner Mongolia autonomous region of China, with a total area of approximately 1.56 × 10^6^ and 1.18 × 10^6^ km^2^, respectively. The MP climate is dry and lacks precipitation due to its long distance from the ocean. The annual average temperature and mean annual rainfall is 4.12 °C and 269 mm, respectively. The MP has a wide variety of regional climates, and most of them are from arid to humid from west to east. All regions are sensitive and vulnerable to drought. The grassland types mainly include grasslands, woody savannas, savannas, and shrub lands in descending order (Figure 9b). 

### 4.2. Data Source and Processing 

We obtained the global land cover maps of 2013 from the MODIS data products (MCD12Q1, http://modis-land.gsfc.nasa.gov/landcover.html/, accessed on 24 February 2020) with the International Geosphere-biosphere Programme (IGBP) classification system. The IGBP classification system defines 17 classes of primary land cover types. In this study, classes 1 to 5 were reclassified as forest, classes 6 to 9 were reclassified as shrubland, classes 12 and 13 were reclassified as farmland, classes 15–16 were reclassified as water, and class 17 was reclassified as city (Table 1).

The 0.05 degree monthly NDVI (normalized difference vegetation index) was offered from the Moderate Resolution Imaging Spectroradiometer (MODIS) data products MOD13C2 (http://ladsweb.nascom.nasa.gov/data/search.html, accessed on 4 March 2020). The 0.05 degree monthly NDII (normalized difference impervious index) was calculated by using a red band 1 from MOD13C2 and thermal infrared band 32 from MOD11C3. Both MOD13C2 and MOD11C3 image datasets were converted to Albers equal area conical projection and WGS-84 datum using the ArcGIS V9.3 software (ESRI, San Diego, CA, USA). To reduce the image noise from the atmospheric clouds, particles, shadows, etc., three-point smoothing was used to improve data quality.

We obtained the monthly meteorological data from the gridded datasets of Climatic Research Unit (CRU) TS 3.22 (http://crudata.uea.ac.uk/cru/data, accessed on 6 January 2019). These gridded datasets cover the global land surface (excluding Antarctica) at a 0.5° resolution and provide the best estimates for month-by-month variations in climate variables [53]. No measurement value is missing in the datasets. The scPDSI datasets were provided by the CRU. The scPDSI uses −0.99–0.99 as normal, and negative values indicate drought. Classification relevant to this research mainly includes extreme moist, heavy moist, moderate moist, slightly moist, slightly normal drought, moderate drought, heavy drought, and extreme drought (Table 2). In order to facilitate spatial statistics, meteorological data are resampled by ArcGIS 10.2 software with a resolution of 0.05 degree.

### 4.3. Methods

#### 4.3.1. Estimation of F_v_

*F*_v_ is an index directly used to determine grassland health condition. We estimated the grassland coverage by using the *NDVI* data due to the significant linear correlation relationship between grassland coverage and *NDVI*. The calculated model is pixel dichotomy model. The specific calculation formula is as follows:

(1) $$F_{v}=\frac{NDVI-NDVI_{\min}}{NDVI_{\max}-NDVI_{\min}}\times 100\%$$

where *F*_v_ is the grassland coverage (%), *NDVI* is the *NDVI* value of a single pixel, *NDVI*_min_ is the NDVI value of bare soil or areas without vegetation coverage, and *NDVI*_max_ is the NDVI value of pixels completely covered by vegetation. Theoretically, the *NDVI*_min_ value should be close to 0, and the *NDVI*_max_ value represents the maximum value of *NDVI* per unit pixel of total vegetation coverage. However, considering the influence of vegetation type, noise, terrain, image quality, and other factors, the *NDVI*_min_ and *NDVI*_max_ values will deviate from the actual values, which are generally represented by the maximum and minimum values within a certain confidence range. In this paper, *NDVI* values near 2% and 98% of the cumulative percentage of *NDVI* values in remote sensing images in the study area are selected as *NDVI*_min_ and *NDVI*_max_ values.

#### 4.3.2. Estimation of F_b_

*F*_b_ is a concept corresponding to *F*_v_. It includes the reflectance and temperature characteristics of the object surface and is not a supplement to *F*_v_. *F*_b_ enriches the research index of grassland ecosystem on the basis of remote sensing technology. In this study, we chose Wang’s estimation formula for *F*_b_ [54], which is expressed as follows:

(2) $$F_{b}=\frac{NDII-NDII_{\min}}{NDII_{\max}-NDII_{\min}}\times 100\%$$

(3) $$NDII=\frac{\lambda_{R}-\lambda_{T}}{\lambda_{R}+\lambda_{T}}$$

where *F*_b_ delineates the surface bareness fractions, *NDII* is the normalized difference impervious index, *NDII*_min_ refers to the minimum value of *NDII* (high grassland coverage and low-temperature pixel), and *NDII*_max_ represents the maximum value of *NDII* (high temperature and reflectivity). *λ_R_* is red band 1 from MOD13C2, and *λ_T_* refers to thermal infrared band 32 from MOD11C3.

#### 4.3.3. Estimation of NPP

*NPP* was extracted from the dataset at a spatial resolution of 1 km and calculated on the basis of the BIOME-BGC model [55]. The specific formula is as follows:

(4) $$NPP=\sum_{t}^{365}PSNet-(R_{m}+R_{g})$$

(5) $$PSNet=GPP-R_{lr}$$

where *NPP* represents the actual *NPP* (g cm^−2^ year^−1^), and *PSNet* refers to the net photosynthesis. R*_m_* is the annual maintenance respiration of live cells in woody tissue, and R*_g_* delineates the annual growth respiration. *GPP* is the gross primary productivity from MOD17A2 datasets, and R*_lr_* is the daily leaf and fine root maintenance respiration. 

#### 4.3.4. Grassland Dynamic Analysis

The grassland vegetation dynamic analysis is a significant ecological process of grassland health condition. We can assess grassland degradation or restoration by using *F*_v_, *F*_b_, and NPP as fundamental indicators. The slope was determined by using ordinary least squares regression, which is expressed as follows:

(6) $$Slope_{A}=\frac{n\times \sum_{i=1}^{n}i\times {\left(A\right)}_{i}-\left(\sum_{i=1}^{n}i\right)\left(\sum_{i=1}^{n}{\left(A\right)}_{i}\right)}{n\times \sum_{i=1}^{n}i^{2}-{\left(\sum_{i=1}^{n}i\right)}^{2}}$$

where *A* refers to grassland *F*_v_, *F*_b_, and NPP; *i* is the sequence number of the year (in this study, 1 is for the year 2000, 2 is for the year 2001, and so on); *n* represents the number of years, which is 14 in this study. A negative slope value shows a degradation trend, whereas a positive slope value shows a restoration trend. In this study, combined analysis of slopes (Table 3) was conducted to quantitatively evaluate the grassland response to drought.

The significance of the variation tendency was determined in terms of *F*-test to represent the confidence level of variation. The calculation for statistics is expressed as follows:

(7) $$F=U\times \frac{n-2}{Q}$$

(8) $$U=\sum_{i=1}^{n}{\left({\hat{y}}_{i}-\bar{y}\right)}^{2}$$

(9) $$Q=\sum_{i=1}^{n}{\left(y_{i}-{\hat{y}}_{i}\right)}^{2}$$

(10) $${\hat{y}}_{i}=Slope\times i+b$$

(11) $$b=\bar{y}-Slope\times \bar{i}$$

where *U* represents the residual sum of the squares; *Q* is the regression sum; ${\hat{y}}_{i}$ refers to the regression value; *y_i_* delineates the average data of year *i*; $\bar{y}$ is the mean value of *F*_v_, *F*_b_ or NPP over *n* years; *b* refers to the intercept of the regression formula. 

We classified the variation tendency into the following six levels on the basis of the *F*-test results: extremely significant decrease (ESD, slope < 0, *p* < 0.01); significant decrease (SD, slope < 0, 0.01 < *p* < 0.05); no significant change (NSC, slope = 0, *p* > 0.05); Significant Increase (SI, Slope > 0, 0.01 < *p* < 0.05); extremely significant increase (ESI, slope > 0, *p* < 0.01).

#### 4.3.5. Correlation Analysis 

The Pearson correlation coefficient was used to reflect the long-term dynamic of two variables in a given time *n*. The specific calculation formula is as follows:

(12) $$r=\frac{n\times \sum_{i=1}^{n}\left(x_{i}\times y_{i}\right)-\left(\sum_{i=1}^{n}x_{i}\right)\left(\sum_{i=1}^{n}y_{i}\right)}{\sqrt{n\times \left(\sum_{i=1}^{n}x_{i}{}^{2}\right)-{\left(\sum_{i=1}^{n}x_{i}\right)}^{2}}\sqrt{n\times \left(\sum_{i=1}^{n}y_{i}{}^{2}\right)-{\left(\sum_{i=1}^{n}y_{i}\right)}^{2}}}$$

where *r* is the correlation coefficient, *n* refers to the sequential year, which is 14 in this study; *x_i_* and *y_i_* represent *F*_v_, *F*_b_ or NPP and climatic factors, respectively. *r* refers to a description of linear correlation degrees between the two variables. The value of *r* ranges from −1 to 1. −1 and 1 are completely related, whereas 0 indicates irrelevant. The greater the absolute value of *r*, the stronger the correlation, but no causal relationship is found. 

## 5. Conclusions

This study investigated the grassland vegetation dynamic on the basis of multi-index and its response to droughts in the MP during 2000–2013 in terms of the relations between F_v_, F_b_, NPP, and scPDSI. The spatial distribution of grassland F_v_ and NPP decreases from northeast to southwest, showing an increasing trend of 0.18 and 0.43, respectively. On the contrary, F_b_ increases from northeast to southwest, presenting a decreasing trend, with the value of −0.16. The grassland degradation condition of the MP shows a restoration trend during the study period. F_v_ and NPP shows a positive relationship with scPDSI, whereas F_b_ is exactly on the country. The areas with a positive correlation between F_v_, NPP, and scPDSI are 84.08% and 93.88%. The grassland increase regions with control and counter response to drought account for 6.06% and 6.87%. However, the distribution of grassland decrease regions with control and counter response (0.44% vs. 0.29%) is minimal. The regions of grassland increase from control response mainly distribute in central MG, whereas the grassland increase regions with counter response are in the eastern and western MG and northeast IM. Such detailed analysis of grassland-related indexes and its responses to drought are useful to clarify the grassland condition, potential effect of drought, and is beneficial to help policymakers for develop proper measures for grassland protection.

## Figures and Tables

**Table 1:** The reclassification of land use type according to the IGBP classification system.

Original Serial Number	Original Land Use Type	Serial Number after Reclassification	Land Use Type after Reclassification
1	Evergreen Needleleaf Forest	1	Forest
2	Evergreen Broadleaf Forest		
3	Deciduous Needleleaf Forest		
4	Deciduous Broadleaf Forest		
5	Mixed Forest		
6	Closed Shrublands	2	Shrubland
7	Open Shrublands		
8	Woody Savannas		
9	Savannas		
10	Grasslands	3	Grassland
11	Permanent Wetlands	4	Wetland
12	Croplands	5	Farmland
13	Cropland/Natural Vegetation Mosaic		
14	Barren or Sparsely Vegetated	6	Desert
15	Snow and Ice	7	Water
16	Water Bodies		
17	City and Built-up	8	City

**Table 2:** Classification relevant of self-calibrating Palmer drought severity index (scPDSI).

scPDSI	Classification of Dry and Wet	scPDSI	Classification of Dry and Wet
≥4	Extreme moist	−1~−2	Slightly drought
3~4	Heavy moist	−2~−3	Moderate drought
2~3	Moderate moist	−3~−4	Heavy drought
1~2	Slightly moist	≤−4	Extreme drought
−1~1	Normally	-	-

**Table 3:** Scenarios to assess the role of F_v_, F_b_ and NPP responding to scPDSI in the MP.

Change Direction	Grassland Conditions	scPDSI	F_v_	F_b_	NPP
control response	Grassland Restoration	Slope > 0	Slope > 0		
		Slope > 0		Slope < 0	
		Slope > 0			Slope > 0
	Grassland degradation	Slope < 0	Slope < 0		
		Slope < 0		Slope > 0	
		Slope < 0			Slope < 0
counter response	Grassland Restoration	Slope < 0	Slope > 0		
		Slope < 0		Slope < 0	
		Slope < 0			Slope > 0
	Grassland degradation	Slope > 0	Slope < 0		
		Slope > 0		Slope > 0	
		Slope > 0			Slope < 0

## Data Availability

Not applicable.
